# Terapia de Anticoagulação com Varfarina: Uma Realidade da Saúde Pública Brasileira que Carece de Estrutura para Melhor Controle

**DOI:** 10.36660/abc.20220504

**Published:** 2022-08-25

**Authors:** Martino Martinelli

**Affiliations:** 1 Hospital das Clínicas Faculdade de Medicina Universidade de São Paulo São Paulo SP Brasil Instituto do Coração do Hospital das Clínicas da Faculdade de Medicina da Universidade de São Paulo, São Paulo, SP – Brasil

**Keywords:** Fibrilação Atrial, Anticoagulantes/uso terapêutico, Acidente Vascular Cerebral, Hemorragia, Tromboembolia, Varfarina/efeitos adversos

O aumento da longevidade, nas últimas décadas, levou ao incremento progressivo da prevalência da fibrilação atrial (FA), em todo o mundo.^1 , 2^

Com isso, a terapia de anticoagulação passou a ser cada vez mais indicada na prevenção de eventos tromboembólicos. A necessidade de uso contínuo dirigiu a preferência para anticoagulantes orais, historicamente representados por fármacos antivitamina K (AVK) e, mais recentemente, por novos anticoagulantes (anti fator X). Dentre esses, a varfarina, um AVK, é o de maior destaque pelo baixo custo.

Entretanto, pelo fato da varfarina ter estreita janela terapêutica, seu uso exige um manuseio visando o equilíbrio entre evitar subdoses que são incapazes de prevenir eventos tromboembólicos e a superdosagem que pode causar eventos hemorrágicos. Esse manuseio da varfarina é dificultado pela enorme variabilidade interindividual de resposta medicamentosa, assim como pelo grande número de interações com outros fármacos e alimentos.^3^

A varfarina está entre os dez medicamentos mais relacionados à ocorrência de erros de dispensação. Nos Estados Unidos e na Austrália, os anticoagulantes orais estão entre as cinco classes mais relacionadas a eventos graves secundários ao uso de medicamentos. ^4^

No Brasil, o Instituto de Práticas Seguras no Uso de Medicamentos (IPSM) inclui a varfarina como droga de alta vigilância, isto é, de uso potencialmente perigoso.^5^ O ajuste da dose ideal de varfarina é monitorado pela Razão Normalizada Internacional (RNI) e a eficiência medicamentosa é estimada pelo tempo na faixa terapêutica (TTR), período de RNI com valores entre 2,0 e 3,0. Existem poucos dados sobre TTR em pacientes com FA na prática comunitária, mas é preciso difundir essa ferramenta cada vez mais.

A taxa de uso da varfarina na rede de saúde pública do Brasil é elevada e o custo-efetividade é controverso.^6^ Existem barreiras práticas sérias para seu uso em nosso meio: baixa aderência causada por limitados recursos financeiros e/ou baixo nível sociocultural, assim como complexidade do manuseio do fármaco pelos profissionais da saúde. Nesse sentido, há evidências de que os médicos brasileiros estão pouco familiarizados com a administração apropriada da varfarina aos pacientes.

Colet et al.,^7^ reportaram o baixo nível de conhecimento de profissionais da saúde pública de um hospital público do estado do Rio Grande do Sul sobre o uso da varfarina. Os autores verificaram que não existe estratégia institucional para tratar do tema e sugerem que os serviços de saúde incluam programas de educação, pelo menos aos mais vulneráveis a eventos adversos, para aumentar a segurança dos pacientes.

Pokorney et al.,^8^ reportaram achados específicos da anticoagulação com warfarina de 5.210 pacientes do registro americano de FA (ORBIT-AF). Ao longo de 18 meses, o TTR médio foi de 65% ± 20% com mediana de 68%. Os pacientes com TTR ≤ 53% eram mais frequentemente do sexo feminino e tinham menos educação universitária do que pacientes com TTR mais elevado. Pacientes com diabetes mellitus, insuficiência renal ou cardiomiopatia também foram menos propensos a apresentar TTR elevado. Entretanto, o achado impactante deste estudo foi a associação de valor de TTR significativamente superior (p <0,0001) entre pacientes assistidos em clínica de anticoagulação (69%) versus assistência ambulatorial geral (66%)

Nesta edição dos Arquivos Brasileiros de Cardiologia, Bazan et al.,^9^ reportam, em estudo realizado em hospital terciário do estado de São Paulo, valor médio de TTR de 52,2% dentre 203 pacientes com FA não valvar. Os autores consideraram esse achado aceitável, associando-o a fatores culturais e socioeconômicos. O manuscrito contém informações valiosas, mas escancara as limitações do nosso sistema público na prevenção de fenômenos tromboembólicos dessa população.

Valores de TTR inferiores a 60% são indicativos de baixa qualidade de anticoagulação. No estudo de Bazan et al.,^9^ 63,5% dos pacientes apresentaram valores de TTR inferiores a 60%, associando essa população a maiores taxas de mortalidade global, sangramento maior, acidente vascular cerebral e tromboembolismo sistêmico.^10^ Os valores médios estimados para países da Europa Ocidental e Canadá/Estados Unidos são respectivamente 63,2% e 64,1%.^10^ Mesmo para América Latina, o valor médio (55,2%)^11^ é superior ao reportado por Bazan et al.^9^

Por outro lado, o achado mais relevante desse estudo, associação entre instabilidade de RNI na fase de adaptação à anticoagulação com maiores taxas de eventos adversos, corroborou a falta de controle do processo global porque correspondeu a valores médios de TTR muito baixos (média de 46,83%).

Então, conclui-se que, para otimizar taxas de sucesso de anticoagulação com varfarina em nosso meio, é preciso criar clínicas de anticoagulação multidisciplinares compostas por médico, farmacêutico, enfermeiro, assistente social e psicólogo.

As clínicas de anticoagulação devem atuar por meio de protocolos assistenciais de manuseio da varfarina pela equipe multidisciplinar, e de programas educacionais dirigidos aos pacientes.

Por fim, é importante destacar que as metas para controle do uso de varfarina pelo nosso sistema de saúde pública devem focar as taxas de eficiência obtidas pelos melhores centros do mundo, tal qual procedemos com inúmeros programas nacionais bem-sucedidos.
