# Supplementary material for: Terapia de Anticoagulação com Varfarina: Uma Realidade da Saúde Pública Brasileira que Carece de Estrutura para Melhor Controle
Source: Arq Bras Cardiol. 2022 Aug 25;119(3):370–1. [Article in Portuguese] doi: 10.36660/abc.20220504 (PMC9438536; doi:10.36660/abc.20220504)
Supplement: Translation

# Translation: Anticoagulation Therapy with Warfarin: A Reality of Brazilian Public Health that Lacks Structure for Better Control

**Authors:** Martino Martinelli

**Affiliations:** 1 Brazil Instituto do Coração do Hospital das Clínicas da Faculdade de Medicina da Universidade de São Paulo, São Paulo, SP – Brazil

**Keywords:** Atrial Fibrillation, Anticoagulants/therapeutic use, Stroke, Hemorrhage, Thromboembolism, Warfarin/adverse effects

The increase in longevity in recent decades has led to a progressive increase in the prevalence of atrial fibrillation (AF) worldwide.^1 , 2^

As a result, anticoagulation therapy has become increasingly indicated in preventing thromboembolic events. The need for continuous use drove the preference for oral anticoagulants, historically represented by antivitamin K (VKA) drugs and, more recently, by new anticoagulants (anti-factor X). Among these, a VKA, warfarin, is the most prominent for its low cost.

However, because warfarin has a narrow therapeutic window, its use requires a balance between avoiding underdoses that cannot prevent thromboembolic events and overdoses that can cause bleeding. This handling of warfarin is hampered by the enormous inter-individual variability of drug response and a large number of interactions with other drugs and foods.^3^

Warfarin is among the ten drugs most related to dispensing errors. In the United States and Australia, oral anticoagulants are among the five classes most related to serious events secondary to medication use.^4^

In Brazil, the Institute for Safe Practices in the Use of Medicines (IPSM) includes warfarin as a high surveillance drug and potentially dangerous use.^5^ The ideal dose of warfarin adjustment is monitored by the International Normalized Ratio (INR), and the drug efficiency is estimated by the time in the therapeutic range (TTR), period of INR with values between 2.0 and 3.0. There are few data on TTR in patients with AF in community practice, but this tool needs to be increasingly disseminated.

The use rate of warfarin in the public health network in Brazil is high, and the cost-effectiveness is controversial.^6^ There are serious practical barriers to its use in our country: low adherence caused by limited financial resources and/or low sociocultural status, as well as the complexity of drug handling by health professionals. In this sense, there is evidence that Brazilian physicians are unfamiliar with the proper administration of warfarin to patients.

Colet et al.^7^ reported the low knowledge of public health professionals at a public hospital in the Rio Grande do Sul about using warfarin. The authors found no institutional strategy to address the issue and suggest that health services include education programs for those most vulnerable to adverse events to increase patient safety.

Pokorney et al.^8^ reported specific findings of warfarin anticoagulation in 5,210 patients from the American AF Registry (ORBIT-AF). Over 18 months, the mean TTR was 65% ± 20%, with a median of 68%. Patients with TTR ≤ 53% were more often female and had less college education than patients with higher TTR. Patients with diabetes mellitus, renal failure, or cardiomyopathy were also less likely to have an elevated TTR. However, the striking finding of this study was the association of TTR values significantly higher (p < 0.0001) among patients seen in the clinic of anticoagulation (69%) versus general outpatient care (66%)

In this issue of *Arquivos Brasileiros de Cardiologia* , Bazan et al.^9^ report that in a study performed in a tertiary hospital in the state of São Paulo, the mean TTR value of 52.2% among 203 patients with non-valvular AF. The authors considered this finding acceptable, associating it with cultural and socioeconomic factors. The manuscript contains valuable information but reveals the limitations of our public system in preventing thromboembolic phenomena in this population.

TTR values lower than 60% are indicative of poor anticoagulation quality. In the study by Bazan et al.^9^ 63.5% of patients had TTR values below 60%, associating this population with higher rates of global mortality, major bleeding, stroke and systemic thromboembolism.^10^ The average values estimated for Western Europe and Canada/United States countries are 63.2% and 64.1%.^10^ Even for Latin America, the average value (55.2%)11 is higher than that reported by Bazan et al.^9^

On the other hand, the most relevant finding of this study, the association between INR instability in the anticoagulation adaptation phase with higher rates of adverse events, corroborated the lack of control of the global process because it corresponded to very low mean TTR values (mean of 46 .83%).

Therefore, it is concluded that to optimize success rates of anticoagulation with warfarin in our country, it is necessary to create multidisciplinary anticoagulation clinics composed of physicians, pharmacists, nurses, social workers and psychologists.

Anticoagulation clinics should operate through care protocols for handling warfarin by the multidisciplinary team and educational programs aimed at patients.

Finally, it is important to highlight that the goals for controlling the use of warfarin by our public health system must focus on the efficiency rates obtained by the best centers in the world, as we have done with numerous successful national programs.
